# Opioids Preconditioning Upon Renal Function and Ischemia-Reperfusion Injury: A Narrative Review

**DOI:** 10.3390/medicina55090522

**Published:** 2019-08-23

**Authors:** Julio Palomino, Raquel Echavarria, Adriana Franco-Acevedo, Bibiana Moreno-Carranza, Zesergio Melo

**Affiliations:** 1 School of Medicine, Universidad Durango-Santander, Hermosillo 83165, Mexico; 2 CONACyT-Centro de Investigacion Biomedica de Occidente, Instituto Mexicano del Seguro Social, Sierra Mojada #800 Col. Independencia, Guadalajara 44340, Jalisco, Mexico; 3 Pharmacology PhD program, Universidad de Guadalajara, Guadalajara 44340, Mexico; 4 School of Medicine, Universidad Anahuac, Queretaro 76269, Mexico

**Keywords:** kidney, ischemia-reperfusion injury, morphine, fentanyl, opioid preconditioning

## Abstract

Kidneys have an important role in regulating water volume, blood pressure, secretion of hormones and acid-base and electrolyte balance. Kidney dysfunction derived from acute injury can, under certain conditions, progress to chronic kidney disease. In the late stages of kidney disease, treatment is limited to replacement therapy: Dialysis and transplantation. After renal transplant, grafts suffer from activation of immune cells and generation of oxidant molecules. Anesthetic preconditioning has emerged as a promising strategy to ameliorate ischemia reperfusion injury. This review compiles some significant aspects of renal physiology and discusses current understanding of the effects of anesthetic preconditioning upon renal function and ischemia reperfusion injury, focusing on opioids and its properties ameliorating renal injury. According to the available evidence, opioid preconditioning appears to reduce inflammation and reactive oxygen species generation after ischemia reperfusion. Therefore, opioid preconditioning represents a promising strategy to reduce renal ischemia reperfusion injury and, its application on current clinical practice could be beneficial in events such as acute renal injury and kidney transplantation.

## 1. Introduction

### 1.1. Essential Concepts of Renal Physiology

The kidneys are involved in several important bodily functions, including blood filtering, resulting in excretion of toxins and metabolic end products, regulating water volume, blood pressure control, secretion of hormones, and acid-base and electrolyte balance [1]. Blood pressure control is directly related to the kidney function. Kidneys maintain adequate blood pressure by regulating sodium and water balance, activation of the renin-angiotensin-aldosterone system and release of endothelin and prostaglandins [2].

Glomerular filtration is one of the major purposes of the kidney. The glomerulus filtrates through three specific layers: The fenestrated endothelium, the glomerular basement membrane, and epithelial podocytes with foot processes [3]. Molecular size and electric charge are two essential factors in glomerular filtration. The most important requirement for kidneys is to preserve a high rate of glomerular filtration through high renal blood flow and oxygen consumption [4]. Oxygen delivery varies throughout the kidney. The renal cortex is considered to be a highly perfused region of the kidney. Much of the renal medulla performs under low PO₂ conditions, specifically the outer renal medulla [4]. This segment is highly susceptible to the hypoxic injury, making it a widely affected area in the ischemia reperfusion injury (IRI) explained below.

Evaluation of the renal function is vital to determine renal conditions in several pathologies. The glomerular filtration rate (GFR) is used to assess the renal function and indicates the amount of fluid filtered by the kidneys, which is dependent on the hydrostatic and osmotic pressure. GFR is equal to the product of the net filtration pressure, hydraulic permeability, and filtration area. Several equations have been established to obtain the GFR such as Cockroft-Gault and MDRD (Modification of Diet in Renal Disease Study Group) equations (Table 1). KDOQI (Kidney Disease Outcomes Quality Initiative) and KDIGO (Kidney Disease Improving Global Outcome Organization) classifies the stage of chronic kidney disease (CKD) based on the GFR estimated by MDRD (see Table 2) [5]. Urinary output is another way to evaluate the renal function. It is the volume of urine produced in an hour depending on the patient’s weight with ≥5 mL/kg/h of urine being the normal value.

Many different conditions can affect the kidney and its function in a short or long term. The most significant pathologies are acute kidney injury (AKI) and CKD. 

### 1.2. Acute Kidney Injury (AKI)

AKI is defined as a reduction in the kidney function with a decreased GFR [6]. It results in: A decrease in urine output (less than 0.5 mL/kg per hour for six hours), an increase of serum creatinine and blood urea nitrogen, and an inappropriate balance of electrolytes [7]. Other abnormalities include pH changes and fluid management alterations. The Acute Kidney Injury Network (AKIN) defines AKI as an abrupt (<48 hours) decrease in the kidney function with an increase in serum creatinine by ≥0.3 mg/dL or ≥50% from the baseline, or a reduction of urine output in less than 0.5 mL/kg per hour for more than six hours [8].

One of the major causes of AKI is ischemia due to partial or total obstruction of the vessels inflow. [9]. Lack of oxygen creates a low energy condition that predisposes to exacerbated oxidative stress and intense inflammation after the restoration of normal blood flow, otherwise known as Ischemia-Reperfusion Injury (IRI). Interestingly, the kidney is one of the most susceptible organs to IRI [10,11]. Segments S2 and S3 of the proximal tubule are vulnerable to oxygen tension changes due to their vast number of mitochondria. Dysregulation of the Na^+^/K^+^ ATPase enzyme and depletion of ATP produce uncoupling of the respiratory chain, free radical production, loss of epithelial cell adhesion, and cell death [12,13,14]. In response to a decrease in renal perfusion, vasodilatation, and vasoconstriction take place by the afferent and efferent arterioles, respectively. Hemodynamic alterations relate to endothelial cell injury resulting in an imbalance of vasoactive substances [15]. Vasodilation is mediated by prostaglandins, bradykinin, and nitric oxide [6,16]. Vasoconstriction is the result of a sympathetic nerve activation, endothelin action, and renin-angiotensin system regulation [17,18,19]. These mechanisms ensure a renal blood flow for an adequate GFR. Other causes of AKI include sepsis, changes in hemodynamic stability, inflammation, nephrotoxicity, and blockage in the passage of urine [6].

One of the most critical mechanisms of AKI is IRI [14]. Kidneys have a high metabolic activity with a high oxygen requirement. The oxygen deficit and nutrient shortage, given by ischemia, result in the loss of cellular adhesion, integrity, and stability. AKI progression involves several sequential phases. The initiation phase starts with the primary ischemic insult, followed by a cascade of necrosis and apoptosis [20]. Clinical and histological manifestations include an increase in serum creatinine, a reduction in urinary volume, a loss of tubule brush order, the formation of tubular casts or dilatation of distal tubule [15]. In the second phase, the damage remains due to inflammation and reperfusion. Then, a release of cytokines takes place accompanied by neutrophil infiltration; stimulating the generation of reactive oxygen and nitrogen species (ROS and NOS), chemotaxis, and phagocytosis [21]. During the third phase, the maintenance phase, GFR is relatively stable, and urinary volume starts to normalize. The last phase is the recovery in which creatinine falls, tubular function increases and renal architecture improves. The types of injury or mechanism causing kidney damage will predict the clinical presentation.

AKI is an entity that if left untreated, can cause irreversible kidney damage, which can progress into CKD or even end-stage renal disease (ESRD). The postoperative development of AKI occurs in 40% of the cases and is related to increased morbidity and high mortality [22,23,24]. The clinical course of postoperative AKI depends on age, comorbidities, and overall health.

Several biomarkers besides creatinine, GFR, and urinary output are currently under study as novel diagnostic approaches for AKI. Examples of these are NGAL (Neutrophil Gelatinase-Associated Lipocalin) and KIM-1 (Kidney Injury Molecule), which are involved in acute responses to injury [25].

### 1.3. Chronic Kidney Disease (CKD)

CKD is a consequence of several chronic diseases such as diabetes or hypertension [26]. Risk factors in developing CKD and nephron loss include male sex, older age, diabetes, proteinuria, hypertension, and hyperuricemia [27]. Nevertheless, a previous episode of AKI can lead to the development of CKD or permanent glomerular damage [28]. The KDIGO and the National Kidney Foundation (NKF) KDOQI guidelines define CKD as abnormalities of kidney structure or function for more than three months or a GFR of <60 mL/min/1.73 m^2^ [29,30]. This damage presents as proteinuria or albuminuria, abnormalities of the urinary sediment or structural abnormalities detected by imaging or histology [31]. The CKD staging can be done whether by GFR or albuminuria category. Based on GFR, CKD is classified in five stages from G1 to G5, also known as ESRD (see Table 2, [29]).

Different pathological conditions affecting the glomerulus, vasculature, or tubulointerstitium can result in kidney structural deterioration. Renal fibrosis is the most common pathological factor in advanced kidney diseases and has shown to be the most reliable predictor of CKD progression to ESRD [32]. The constant insult that causes CKD activates a profibrotic state mediated by myofibroblasts. As a result, there is a tubular cell loss along with collagen deposits [33]. Fibrosis is the result of excessive accumulation of extracellular matrix (ECM) components such as collagen and fibronectin secreted by myofibroblasts in response to cell damage [34]. The formation of the fibrotic tissue is the final manifestation in diverse renal pathologies such as glomerulosclerosis, tubular atrophy, and interstitial fibrosis [26]. These entities are characterized by prolonged tissue insult, inadequate repair mechanisms, fibroblasts activation, and macrophage infiltrates.

Signaling pathways involved in renal fibrosis are complex and not completely elucidated. Several mechanisms underlying fibrogenesis have debuted, all of them with important characteristics that result in a multifaceted process. Representative molecules that participate in fibrosis are hypoxia-inducible factor 1, transforming growth factor-beta, nuclear factor-kappa b (NF-κb), angiotensin II, reactive oxygen species (ROS), interleukin 6 (IL-6), interleukin 8 and kidney injury molecule 1 [32].

Patients that develop CKD exhibit numerous factors that contribute to the decline of the kidney function and the following clinical symptoms as hypertension, proteinuria, and mineral misbalance. Progression to ESRD is almost inevitable and chronic inflammation appears to be the main trigger. Macrophages and the interstitial fibroblast support the production of cytokines and collagen that modify and accumulate in the extracellular matrix. Finally, pharmacological approaches are minimal and based on controlling symptoms, leaving replacement therapies such as dialysis and transplantation as the best options to patients suffering from ESRD.

### 1.4. Transplantation

Kidney transplant is considered one of the most suitable treatments for ESRD patients. However, during the transplant surgery, there is a cessation of renal blood flow with a posterior reestablishment, and therefore, the IRI phenomenon always takes place. Other conditions, such as trauma or vascular and heart surgery also cause IRI. Hypoxia and nutrient deprivation due to ischemia results in an excessive generation of ROS, which cause cell death and inflammatory responses [35]. Biochemical changes in cells include suppression of oxidative phosphorylation, ATP reduction, activation of anaerobic respiration, and inhibition of the Na^+^/K^+^ ATPase pump [36]. The organism answers with the production of cytoprotective molecules. Reperfusion, consisting of a reinstitution of blood flow, confers a second wave of cellular damage after ischemia. Tissue injury increases as a result of generalized inflammation and the activation of harmful cell responses. ATP production normalizes due to aerobic metabolism initiation, but reoxygenation causes a rise in ROS formation. Both the superoxide anion and hydrogen peroxide induce hydroxyl radicals and disturb the cell membrane integrity [37].

The IRI mechanism involves the activation of the immune system by neutrophils, macrophages, and dendritic cells in kidneys. Endothelial and tubular epithelial cells also play an essential role in the inflammation induced by IRI [38,39]. Ischemia affects tubular epithelial cells by damaging their structure and function; and leads to apoptosis, necrosis, and interstitial inflammation. Hypoperfusion induces endothelial cell swelling, capillary deterioration, increased permeability, and expression of intracellular adhesion molecule-1 (ICAM-1) and E- and P-selectins [35,40,41]. The ischemic process liberates compounds from the injured tissue such as fibronectin, heat shock proteins, and DNA that activates toll-like receptors (TLRs). TLRs enable the production of proinflammatory cytokines such as tumor necrosis factor-alpha (TNF-α), interleukin-1 beta (IL-1β), and IL-6 through NF-κb activation [42].

Kidney injury is worsened by the aggregation of neutrophils to endothelium in tubular capillaries and kidney interstitium. Neutrophil-endothelium adhesion causes neutrophils to activate, release their granules and secrete proteases, which generate ROS. That same activation produces interferon-gamma (INF-γ), interleukin 4 (IL-4), IL-6, interleukin 10 (IL-10), and TNF-α secretion [43]. Neutrophils are the first cells to be recruited and to cause injury in the reperfusion setting [44]. Macrophages also promote IRI by releasing chemokines and proinflammatory cytokines such as IL-1β, IL-6, interleukin 12 (IL-12), and TNF-α [45]. Additionally, dendritic cells activate the natural killer T cells which produce INF-γ, stimulate macrophages, and intensify the immune response. Both macrophages and dendritic cells enable sterile inflammation after reperfusion, and their renal infiltration following transplantation is involved in delayed graft function and acute rejection [46].

## 2. Preconditioning

Preconditioning refers to the molecular changes that occur at the tissue level, which enable that same tissue to adapt and overcome later adverse events. Preconditioning includes the physiological and molecular adaptations in a changing environment. As a minor event, ischemic preconditioning is an example of how the body uses adverse conditions to improve tissue response for following incidents. Ischemic preconditioning is an adaptive mechanism that takes place in multiple organs like the brain, heart, kidney, liver, and muscle.

Brief intervals of ischemia and early reperfusion have been considered as a beneficial therapeutic approach to contain the damage caused by further and more prolonged episodes of ischemia [47]. Protective effects have been observed when tissue preconditioning was used to prepare organs before surgery or pathological insults [48]. For example, in a dog model, several short episodes of ischemia and reperfusion before causing a more extended ischemic event resulted in a protective memory effect in myocytes [49].

Preclinical studies have shown evidence about renal protection conferred by a preceding ischemic event. Classical articles demonstrated that the ischemic preconditioning could reduce IRI improving the renal function, metabolic homeostasis, and preserving cell integrity and tissue morphology. However, they do not leave clarity about the mechanism involved [50,51,52]. Recent evidence confirms the role of ischemic preconditioning upon the renal function. This procedure influences the ROS production and lipid peroxidation through the engagement of antioxidant enzymes [53]. Another suggested mechanism through which ischemic preconditioning attenuates damage is the inhibition of the NF-kβ pathway, reducing inflammatory responses [54].

The potential clinical use oriented to prevent AKI has already been tested in clinical trials. This strategy is able to reduce the incidence of AKI in patients undergoing a cardiovascular surgical procedure [55]. However, it is necessary to contemplate the difficulty of assessing the isolated effect of ischemic preconditioning in patients undergoing a surgical procedure; considering all factors involved (anesthesia, surgical procedure, baseline disease). Overall, ischemic preconditioning increases the probabilities of a good clinical prognosis after a high-risk surgery [56,57,58,59].

The molecular responses caused by ischemia and reperfusion can be reached pharmacologically with different anesthetic substances [60,61]. This is known as anesthetic preconditioning. Experimental and clinical data have shown that anesthetics have protective effects in several organs against IRI [62]. Back in 1976, Bland et al. used halothane as anesthetic preconditioning in dogs to reduce myocardial ischemia [63]. Over the years, preconditioning with anesthetics has changed with a variety of drugs including inhaled and injectable agents. Barbiturates have been used for neuroprotective strategies aiming for a reduction in the ATP consumption in brain tissue. However, no conclusive evidence has been provided for this anesthetic method of protection [64]. Volatile anesthetics like sevoflurane and isoflurane have also been tested trying to demonstrate beneficial results of their usage [65]. Propofol and opioids are other examples of anesthetic agents used to achieve preconditioning with protective effects; we will review them in more detail below.

In summary, tissue exposure to an ischemic condition can enable cells to adapt rather than suffer damage. Therefore, cells under those conditions are able to manage further challenging situations more effectively.

## 3. Opioids

A comprehensive understanding of the nature of opioids and its influence on kidney pathophysiology may improve morbidity and mortality in patients undergoing surgical procedures as transplantation, and consequently, has the potential to modify clinical practice. Furthermore, opioids are commonly used to manage pain in CKD and post-transplant patients [66].

Back to the 1800s, the first known opioid (morphine) was isolated from opium [67]. Four natural alkaloids can be isolated from opium: Morphine, codeine, papaverine, and thebaine. Semisynthetic compounds include diamorphine (heroin), buprenorphine, and oxycodone. Fentanyl, methadone, sufentanil, and remifentanil are examples of synthetic compounds [68]. Another way to categorize opioids is by the receptors in which they have effects. They can be agonists, partial agonists or antagonists. Opioid receptors are G-protein coupled receptors, and the three central receptors are μ, κ, δ (mu, kappa, and delta). The Mu receptor activation causes analgesia, sedation, respiratory depression, bradycardia, and nausea. Spinal and supraspinal analgesia is achieved by a delta receptors activation. Kappa receptors produce spinal analgesia, diuresis, and dysphoria. All three receptors are distributed in the central and peripheral nervous system as well as the gastrointestinal tract. Endogenous peptides with similar effects as opioids can be found naturally in our body. They are called enkephalins and interact with opioid receptors, just like opioids do [69].

Analgesia mediated by opioids is induced by binding to μ receptors in GABAergic neurons. These neurons inhibit descendent neurons in the brainstem and produce analgesia. The same effect is also obtained by inhibiting the release of pain mediators such as substance P, nitric oxide, and glutamate [70]. Along with analgesia come emotional changes caused by opiates probably by the high concentration of opiate receptors in the limbic system. The solitary tract, an area controlling the respiratory activity, has abundant μ2 subtype and δ receptors explaining the changes in the respiratory pattern related to opioids [71]. Constipation is a common side effect of opioid administration. The μ and κ receptors activation in the small and large intestine increases the resting tone of the intestine itself and also the sphincter resulting in decreased peristalsis [72].

Opioid receptors can also be found in the kidneys, with the δ receptor broadly expressed and, on a smaller scale, the κ type [73]. Although its distribution and function in the kidney have not been fully elucidated, some studies propose that the κ receptor activation stimulates activities such as the proliferation of mesangial cells [74]. Additionally, evidence suggests that activation of the κ receptor protects the kidney from IRI through the PI3K/AKT pathway [75].

### 3.1. Morphine

Morphine is known as the opioid prototype to which other opioids are compared. Both μ and δ receptors bind morphine to have its effect. These receptors are widely distributed in the human brain, mainly in the amygdala, hypothalamus, thalamus, and several cortical areas. Morphine has two major metabolites, morphine-3-glucuronide (M3G) and morphine-6-glucuronide (M6G, 60% of metabolites). M6G has been known to have a higher analgesic effect compared to M3G [76]. Morphine has been used in the treatment for acute or chronic pain, and also in the pain management of myocardial infarction. Its administration is variable being intravenously, orally, and subcutaneously. It is metabolized in the liver and excreted in the urine in 72 h after administration. Its maximum effect is reached in 20 min with an approximated duration of three to seven hours. Decreased respiratory effort, low blood pressure, somnolence, vomit, and constipation are expected side effects of morphine.

Undoubtedly, the analgesic effects of morphine are supported by abundant scientific and clinical evidence. However, the properties on non-nervous tissues such as the kidneys are poorly understood and controversial. Some reports show that morphine has antioxidant properties and is a potent modulator of immune responses [77,78]. Other reports showed that morphine is capable of enhancing the severity of damage in a model of nephrotoxicity by cisplatin [79]. Meanwhile, a different study revealed that the prolonged use of morphine is associated with renal dysfunction in tumor-bearing mice [80].

### 3.2. Fentanyl

Fentanyl was developed in 1960 by Paul Janssen as a synthetic opioid drug for pain treatment and anesthesia. The range of effects consists of analgesia, anxiolysis, euphoria, and drowsiness [81]. It works by full agonism of μ receptors and has approximately 50–100 times more potency than morphine. This potency can be explained by its high lipophilicity, which permits a rapid diffusion through cell membranes, the capability of crossing the blood-brain barrier, and high μ receptor affinity. The elimination half-life is around 219 min conferring a protracted effect [82]. Pain threshold is increased in the central nervous system by inhibition of ascending pathways. Differences in dosage administration will make variations of clinical effects. Concentrations of 0.3–0.7 ng/mL will cause an analgesic effect while concentrations higher than 3 ng/mL can represent respiratory and central nervous system depression [83]. Common adverse effects include nausea, constipation, pruritus, orthostatic hypotension, urinary urgency or retention, dry mouth, asthenia, hallucinations, depression, and dyspnea. Fentanyl elimination is through biotransformation in the liver into norfentanyl and is excreted in the urine.

Although much has been studied about the beneficial properties of fentanyl in vivo and in vitro in the heart and cardiomyocytes [84,85,86], little is known about the potential protective properties of fentanyl in renal IRI. However, clinical evidence suggests that its use may be beneficial, and some authors consider that the fentanyl use may be a safe option in patients undergoing hemodialysis and in renal transplant recipients [87,88].

## 4. Opioid Preconditioning and Kidney Protection

There is considerable research interest in potential methods of renal protection against IRI. Proof from experimental studies shows that anesthetic preconditioning with volatile agents can be achieved and protect the heart, brain, and kidney from IRI [89]. Several studies in cardiac surgery have reported improved myocardial mechanical function and reduced myocardial infarct size with volatile anesthetics as preconditioning [90]. Another anesthetic mechanism by which preconditioning can be obtained is with opioids. Opioid preconditioning is a phenomenon that results from an intervention mediated with opioids before an ischemic insult, and that concludes in a reduction of the affected tissue. The morphine cardioprotective effect is conferred by the activation of the δ receptor-KATP channel-linked mechanism [91]. There is abundant literature related to opioid-mediated cardioprotection, though information about the effects of preconditioning on the kidney is limited.

Experimental renal ischemia and reperfusion in rabbits showed that morphine significantly inhibited superoxide generation by neutrophils, suggesting a potential for reducing the oxidative stress after hypoxia [92]. Habibey et al. demonstrated that morphine has also a protective effect in kidneys. The renal function (serum creatinine and BUN) was preserved after 45 min of ischemia and 24 h of reperfusion, causing a marked ischemic tolerance of the kidneys [93]. Preconditioning studies have been made in rats in which opioids induce renal protection, which can be diminished with naloxone [94]. This same research group tested low doses of morphine in an IRI model, concomitantly with three other medications, to find clinically safe and non-toxic doses of morphine. The results showed that all the doses used (20 and 30 mg kg/day per five days) resulted in reduced IRI [94]. Morphine and fentanyl may well lessen the caspase 3 activation induced by ischemia. These opioids delivered improved kidney tubular cell protection when administered before ATP depletion using in vivo and in vitro models [95].

Morphine, fentanyl, and other opioid medications are strong analgesics used frequently in analgesia and pain management, despite reported concerns about drug safety. Long-term administration is commonly associated with the development of side effects including tolerance, dependence, and addiction. Therefore, it is necessary to discriminate between the long-term effects and the effects of single uses in a surgical procedure and preconditioning. Pain management is an essential part of the comprehensive care of patients with CKD at any stage [66]. One of the most used analgesia options in patients with CKD are opioids [96]. Following special considerations, such as dose and exposure time depending on the patient conditions and nature of pain [66,96]. However, prolonged use has been associated with albuminuria and alterations in renal markers indicating kidney dysfunction [97]. This information correlates with findings from animal studies where morphine is found responsible for the development of albuminuria through altering the filtration barrier and negatively influencing the integrity of podocytes [98]. Studies from Lentine et al. showed that the level of use of opioids increased significantly the risk of post-transplant complications [99]. Similarly, a recent study reports that a high rate of long-term opioid prescription in the prevalent kidney transplant population associates with an increased risk of mortality and graft loss [100].

Complex surgical anesthesia schemes in humans make it difficult to dissect the effect of these drugs on the renal function; however, perioperative use showed positive results. This is how the study by Terashi et. al. showed that anesthetic management using remifentanil exerted a renal protective outcome in perioperative adult patients with CKD for at least two weeks after orthopedic surgery [101]. Conversely, different outcomes were obtained when opioids were used after the transplantation surgery. Apparently, there is no association between the chronic opioid use and graft dysfunction [102].

## 5. Extra-Renal Opioid Preconditioning Mechanisms

Actions of opioid drugs in the kidney have not been well characterized. Nonetheless, most of the information about mechanisms involved in organic protection conferred by opioids come from studies in organs as heart and brain. We will summarize below some of the molecules involved considering the overlapping molecular mechanisms between the kidneys and other organs it is important to mention.

Experimental and clinical studies showed that opioids can positively influence the cardiac function and could reduce the size of an infarction resulting from prolonged ischemia [103]. Opioids participate in a reduction of damage to myocardial intracellular structures, a decrease in the dysfunction of the cardiac contractile machinery, and a direct reduction in arrhythmias [104]. Opioids activate the δ and k receptors coupled to the G_i_ proteins and also can activate the µ receptors, but this has very low expression in cardiomyocytes [105]. The δ receptor is most important in the preconditioning phenomena, and this defense can be abolished using the pharmacological antagonist naloxone [104,106]. The δ receptor activation may lead to protection through a reduction of the inflammatory response comprising neutrophil activation. Meanwhile, activation of the k receptors directly participates in the stimulation of the NOS/NO signaling pathway [107] and involve protein kinases pathways as AKT/Pi3K and ERK/MAPK [108]. Morphine or remifentanil administration before myocardial IRI can induce cardioprotection through the µ receptors via the ERK/GSK-3b signaling pathway [109]. This receptor has also been postulated as a potential therapeutic target for opioid-induced protection during heart failure [105,109].

Fentanyl, morphine, and remifentanil are frequently used for neurosurgical procedures. Effects on the brain and nervous system undergoing an ischemic event have been documented in experimental models [110]. Preconditioning with morphine protects neurons against IRI. This effect is mediated by the increased activity of the mammalian Target of Rapamycin, mTOR, pathway resulting in a reduction of oxidative stress and apoptotic agents [111]. Additionally, recent studies suggested that morphine could protect the brain from ischemia, decreasing pro-apoptotic molecules production [112]. Similarly, remifentanil suppresses the apoptotic pathways blocking the association of TNF-α to its receptor [113]. Opioids are also strongly related to the protection from IRI in other tissues such as the liver [114,115], endothelium [116] and skeletal muscle [117,118] and involve similar mechanisms of action.

## 6. Other Anesthetics in IRI

Effective and safe anesthesia for successful transplantation depends on an understanding of the influence and interactions with other anesthetics [119]. Here, we mention some of the most commonly used in kidney transplant and the effects upon IRI.

Propofol is an intravenous drug widely used in anesthesia as an inducer. It is characterized by its rapid onset of action and the speedy recovery of the patient from its effects. It has been shown to have a protective effect at the cardiac and renal level in reperfusion ischemia models [120], this effect is mediated by various mechanisms, mainly due to the scavenging of oxidative species [121,122,123]. Li et al. demonstrated in an IRI experimental model in rats that the administration of propofol previous to ischemia results in lower levels of creatinine, urea, myeloperoxidase, and malondialdehyde, as well as, lower expression of pro-apoptotic proteins. They also reported increased levels of superoxide dismutase, concluding that propofol prevents IRI via inhibiting the oxidative stress pathway [124].

Dexmedetomidine is an alpha-adrenergic receptor agonist drug and extensively used as a sedative. Dexmedetomidine protects against myocardial infarction [125], ischemic brain [126] and kidney injury [127]. Previous studies have shown that preconditioning with Dex in rats has a protective effect on the renal function after I-R through the inhibition of the Janus kinase/signal transducers and activators of the transcription (JAK/STAT) signaling pathway [128]. According to Lempiainen et al. dexmedetomidine preconditioning ameliorates renal IRI and inflammatory response, at least in part, through the p38-MAPK pathway [127].

Isoflurane is a common volatile anesthetic in the clinic and protects against ischemic brain injury [129] by suppressing apoptosis [130]. Preconditioning using a clinically relevant concentration of isoflurane can attenuate renal IRI. These protective effects are mediated by its ability to control inflammation and apoptosis [131]. Additionally, desflurane preconditioning associated with a reduction in IRI, preserving the micromorphology of the kidney [132]. By contrast, some volatile anesthetics can promote IRI. This is the case of halothane. In an experimental model of liver IRI, halothane was shown to stimulate the release of hepatocellular enzymes, indicating an increase in damage [133].

Finally, barbiturates can also reduce the severity of IRI in cardiac models [134]. The molecular mechanisms in renal physiology remain to be elucidated.

## 7. Conclusions

Potential new uses of old well-known drugs as opioids is an emerging field for kidney research. Anesthetic preconditioning is a promising strategy to reduce renal IRI, and its application on current clinical practice could be beneficial in events such as acute renal failure and kidney transplantation. Current evidence suggests that opioids provide organ protection by decreasing reactive oxygen species and inflammation. However, more experimental evidence is still needed to understand the physiological and molecular mechanisms involved in the protection of the kidney and to translate our current knowledge into clinical settings.

## Figures and Tables

**Table 1:** Glomerular filtration rate (GFR) formulas.

GFR Estimation	Formula
Cockroft-Gault Formula	$\mathrm{CrCl}\;\left(\mathrm{mL}/\min\right)=\frac{140-\mathrm{age}\times \mathrm{Lean}\;\mathrm{Body}\;\mathrm{Weight}\left(\mathrm{kg}\right)}{\mathrm{Scr}(\mathrm{mg}/\mathrm{dL})\times 72}\left(\times 0.85\;\mathrm{if}\;\mathrm{female}\right)$
MDRD Formula	$\mathrm{GFR}\left(\mathrm{mL}/\min/1.73m^{2}\right)=\;186\times \mathrm{SCr}{\left(\mathrm{mg}/\mathrm{dL}\right)}^{-1.154}\times {\mathrm{age}}^{-0.203}\times 0.742\left(\mathrm{if}\;\mathrm{woman}\right)\;\;\;\;\;\;\;\;\;\;\;\;\;\;\;\;\;\;\;\;\;\;\;\times 1.21\left(\mathrm{if}\;\mathrm{Black}-\mathrm{American}\right)\;$

CrCl: Creatinine clearance, mL: Milliliters, min: Minutes, kg: Kilograms, SCr: Serum creatinine, mg: Milligrams, dL: Deciliters, m: Meters.

**Table 2:** Classification of the glomerular filtration rate (GFR) stages used by KDOQI (Kidney Disease Outcomes Quality Initiative) and KDIGO (Kidney Disease Improving Global Outcome Organization).

Stage	Classification	GFR Range
G1	Normal or high	≥90 mL/min/1.73 m^2^
G2	Mildly decreased	60–90 mL/min/1.73 m^2^
G3a	Mildly to moderately decreased	45–59 mL/min/1.73 m^2^
G3b	Moderately to severely decreased	30–44 mL/min/1.73 m^2^
G4	Severely decreased	15–29 mL/min/1.73 m^2^
G5	Kidney failure	<15 mL/min/1.73 m^2^

mL: Milliliters, min: Minutes, m: Meters.
